# Bayesian optimisation for breeding schemes

**DOI:** 10.3389/fpls.2022.1050198

**Published:** 2023-01-11

**Authors:** Julien Diot, Hiroyoshi Iwata

**Affiliations:** Department of Agricultural and Environmental Biology, Graduate School of Agricultural and Life Sciences, The University of Tokyo, Tokyo, Japan

**Keywords:** Bayesian optimisation, breeding scheme, genomic selection, computer simulation, genetic simulation, breedSimulatR

## Abstract

**Introduction:**

Advances in genotyping technologies have provided breeders with access to the genotypic values of several thousand genetic markers in their breeding materials. Combined with phenotypic data, this information facilitates genomic selection. Although genomic selection can benefit breeders, it does not guarantee efficient genetic improvement. Indeed, multiple components of breeding schemes may affect the efficiency of genetic improvement and controlling all components may not be possible. In this study, we propose a new application of Bayesian optimisation for optimizing breeding schemes under specific constraints using computer simulation.

**Methods:**

Breeding schemes are simulated according to nine different parameters. Five of those parameters are considered constraints, and 4 can be optimised. Two optimisation methods are used to optimise those parameters, Bayesian optimisation and random optimisation.

**Results:**

The results show that Bayesian optimisation indeed finds breeding scheme parametrisations that provide good breeding improvement with regard to the entire parameter space and outperforms random optimisation. Moreover, the results also show that the optimised parameter distributions differ according to breeder constraints.

**Discussion:**

This study is one of the first to apply Bayesian optimisation to the design of breeding schemes while considering constraints. The presented approach has some limitations and should be considered as a first proof of concept that demonstrates the potential of Bayesian optimisation when applied to breeding schemes. Determining a general "rule of thumb" for breeding optimisation may be difficult and considering the specific constraints of each breeding campaign is important for finding an optimal breeding scheme.

## 1 Introduction

Development of new genotyping technologies has provided breeders with access to genotypic values of several thousand genetic markers in their breeding material. This information along with phenotype data, has allowed breeders to estimate the effects of these markers on the phenotypic traits of interest and to assess the genetic value of their breeding population. Thanks to prediction models, breeders can now perform genetic selection (GS), which involves selection of un-phenotyped individuals based on their genotype data (Meuwissen et al., 2001).

Although using genomic selection can help breeders, it does not guarantee efficient genetic improvement. Therefore, other factors need to be considered when designing breeding schemes. For example, selecting only those individuals with the highest predicted values (Meuwissen et al., 2001) can be interesting for short term breeding whereas other methods like weighting the marker effects by the allele frequency (Jannink, 2010) might yield better results in long term breeding.

Other factors influencing the performance of breeding schemes include the constraints faced by breeders, including straightforward examples such as the budget allowed for the breeding campaign, the initial population, the genotyping or phenotyping capabilities, and so on. Breeders should not neglect these constraints when optimizing a breeding scheme as the optimal decisions made under particular constraints (e.g., a large budget with highly heritable target traits) may differ under different constraints (e.g., low budget and low heritable target traits).

Several examples of methods for optimizing breeding improvement have already been reported, including weighted genomic selection (Jannink, 2010), optimal haploid value (Daetwyler et al., 2015), expected maximum haploid breeding values (Müller et al., 2018), look-ahead selection (LAS) (Moeinizade et al., 2019), and complementarity-based selection (CBS) (Moeinizade et al., 2020). These methods, except LAS and CBS, which also consider the breeders’ budget, mainly focus on the selection criteria for improving the breeding schemes; however this is only one of their aspects. Multiple components of breeding schemes may affect efficiency (Henryon et al., 2014), including the breeding objective definition, available infrastructures, genotyping and phenotyping strategies, prediction models, and selection and mating strategies. All these components, which interact with each other, should be optimised together to obtain a well-designed breeding scheme (Henryon et al., 2014).

In this paper, we introduce the Bayesian optimisation method (Jones et al., 1998). Bayesian optimisation is a specific optimisation method suitable for determining the optimum of a black box function, the “objective function”, whose evaluation has a high cost (e.g., time consuming, financially expensive, limited opportunity, etc.). The main principle behind this method is to fit a Bayesian model, often a Gaussian process, to obtain the posterior distribution of the objective function, and then use this distribution to sample the objective function at the points which are the most promising to be the global extremum. Bayesian optimisation has already been used in several domains like chemistry (Burger et al., 2020), and others (Shahriari et al., 2016); however, very few examples are noted in breeding, such as (Tanaka and Iwata, 2018) and (Hamazaki and Iwata, 2022).

In this study, we present the use of Bayesian optimisation to optimise breeding schemes under specific constraints faced by breeders. We consider the function associated with the breeding scheme outcome as the objective function. However, realizing actualbreeding campaigns to evaluate this objective function would have required several years of experimentation. Therefore, to evaluate the objective function in a reasonable time, like most studies presented above (Jannink, 2010; Daetwyler et al., 2015; Müller et al., 2018; Moeinizade et al., 2019; Moeinizade et al., 2020; Hamazaki and Iwata, 2022), the breeding process has been simulated computationally.

## 2 Materials and methods

### 2.1 Optimisation problem

In this section, we explain the relationship between the breeding scheme and optimisation. For simplicity, we will only consider one phenotypic trait of interest here. In case of multiple traits, a function that maps the value of each trait to a selectionindex value (e.g., the weighted sum of these traits) can be used for generalisation, and the selection index can be considered the target trait. After a breeding campaign, the mean genotypic values for this trait over the individuals in the final population can be expressed as:


(1) u=f(x)+ϵ

Where *x* is the value of all the parameters representing the breeding scheme, *f* (*x*) is the expected genotypic value of the population under the parameter values *x* and *ϵ* is the residual due to the randomness of the breeding process.

Let *X* be the domain of all possible breeding schemes. A breeding scheme can be parameterised by a very large number of variables. In this paper, we consider the following parameters for representing the breeding scheme:

*X =* [ B , C *_p_*, C *_n_*, N *_gen_*,*Pop_init_*,*i_init_*, *i*, *B_rep_*, *pheno_p_*]

where:

-
B : total budget for the breeding campaign
-
C *_p_*: cost for phenotyping one plot
-
C *_n_*: cost for generating and genotyping one new individual in a breeding population
-
N *_gen_*: total number of selection cycles (i.e., generations) in the breeding campaign
-
P *op_init_*: genotypes of the initial population (homozygote individuals)
- *i_init_*: selection intensity for the initial generation (homozygote individuals)
- *i*: selection intensity for all later generations (heterozygote individuals)
- *B_rep_*: part of the total budget allocated for phenotyping; the rest will be used for generating new individuals
- *pheno_p_*: period of phenotyping experiments. Individuals will be phenotyped every *pheno_p_* generations

This parametrization separates the selection intensity of the first and later generations *i_init_* and *i*, respectively. As explained in section 4.3.4, the number of individuals in the second generation is directly related to the selection intensity used on the initial population, and the size of the first generation may have an impact on the performance of the breeding scheme.

Here, we assume that *u* is the target of selection and that a larger *u* is preferred. As breeders may not have control over the first five parameters or over *ϵ* , the best breeding scheme that considers the constraints can be expressed as follows:


(2) $$z^{*}=\arg\;\max{\;}_{z\in Z}g(z)$$

where *Z*=[*i*_*init*_,*i*,*B*_*rep*_,*pheno*_*p*_] and *g*:*z*↦*f*(*z*, B , C _*p*_, C _*n*_, N _*gen*_, P *op*_*init*_) with B , C *_p_*, C *_n_*, N *_gen_*, P *op_init_* are the respective values taken by the variables *ℬ*, *𝒞*_*p*_, *𝒞*_*n*_, *𝒩*_*gen*_, *𝒫op_init_* under the breeder’s constraints.

To optimise this objective function, we used the Bayesian optimisation method using Gaussian process regression at the estimation step, and the value of the objective function was returned by the breeding simulation algorithm.

### 2.2 Bayesian optimisation

Bayesian optimisation consists of a three-step cycle, starting with some observed values of the objective function:

- Bayesian analysis step: Calculation of the posterior distribution of the objective function using all the observed values of the objective function (i.e. the training data).
- Sampling step: Selection of the following sampling points that maximize the acquisition function. The acquisition function is a computationally easy function that can be evaluated at any point of the research space using the posterior distribution of the objective function, as well as return how much evaluating the objective function at this point would help yield the global maximum.
- Evaluation step: Evaluation of the objective function at the previously selected sampling point.

These steps are repeated until the stopping criterion is satisfied.

Our Bayesian analysis is based on a Gaussian process. The Gaussian process is a collection of random variables, in which any finite number has a joint Gaussian distribution. In our case, the random variables represent the values of the objective function *g* (*z*) over parameter space *Z*. The Gaussian process can be fully specified by its mean function *m*(*z*)=*E*[*g*(*z*)],∀*z*∈*Z* and its covariance function, also called a kernel, *k*(*z*,*z* ^′^),∀*z*,*z* ^′^∈*Z* ^2^ (Rasmussen and Williams, 2006). In this study, the following Gaussian kernel was used:


$$k\left(z,z^{\prime }\right)=\exp\;(\frac{-d^{2}}{2\theta^{2}})$$

where *d* is the Euclidean distance between the scaled values of *z* and *z*′ , and *θ* is a hyperparameter estimated using the maximum likelihood method.

For the sampling step, to use the parallel capabilities of modern computers, we sampled *q* evaluation points. This was done using a combination of expected improvement (EI) and a constant liar strategy (Ginsbourger et al., 2007) as acquisition function.

The EI, one of the most well-known acquisition functions for Bayesian optimisation (Jones et al., 1998), is the probability of improvement weighted by the value of the improvement. It can be expressed by


(3) $$EI\left(z\right)=E\left[\max\;(0,g\left(z\right)-{\hat{g}}_{max}\right)]$$

where ${\hat{g}}_{max}$ is the maximum mean predicted value of the objective function among the training data (i.e., the existing sample points). EI is easy to compute and can be optimized using the *Focus Search* algorithm described in Bischl et al. (2017).

To select the *q* sampling points *z*_*j*_, *j*∈{1, *q*}, the EI was iteratively maximised while updating the Gaussian process model using *g*(*z*_*k*_) = *L*, ∀*k *< *j*+1 (Ginsbourger et al., 2007) with *L* = *g_min_* the minimum observed value of the objective function was used among the training data. The objective function for the next Bayesian optimisation iteration was then evaluated in parallel on *q* cores over the selected *z*_*j*_, *j*∈{1, *q*}.

Moreover, to avoid stucking the optimisation at one point by sampling the same points several times, *z*_*j*_, *j*∈{1, *q*} were filtered according to their distances from the existing sample points in the training data. If this distance was less than the threshold *filter_tol_*, the corresponding point was replaced by a randomly selected point in the parameter space.

Finally, the optimisation can be completed after it reaches a specified number of iterations *n_iter_*.

Once the optimisation was completed, we used the latest Gaussian process model to predict the value of the objective function for all visited points. The algorithm returned the point with the highest predicted value based on the Gaussian process model as a result of optimisation.

To evaluate the potential of this method, another naive optimisation algorithm was implemented, in which the *q* sampled points were selected randomly in the parameter space. In this paper, we call this method “random optimisation.” This optimisation returns the point with the highest value calculated using the objective function.

#### 2.2.1 Optimisation results evaluation

To compare the optimised breeding schemes, we independently simulated *n_evaluation_* breeding campaigns after each optimisation, according to the optimal breeding scheme parameters. We obtained *n_evaluation_* samples of the optimised breeding schemes. These results can be used to compare the breeding schemes resulting from each optimisation method.

### 2.3 Stochastic breeding simulations

#### 2.3.1 Overview of the breeding process

For simulating the breeding process, it is easier to use a different parametrisation than what we used for the optimisation. Specifically, we introduced the parameters *n_P_*t*__*, the number of phenotyping plots available for the generation *t*, *n_S_*t*__*,the number of selected parents in the generation *t*, and *n_new_*t*__* the number of progenies to generate at the generation *t*. All those parameters derived from the optimisation’s parameters and constraints. Their calculation are described in section 4.3.4.

**Figure 1:**
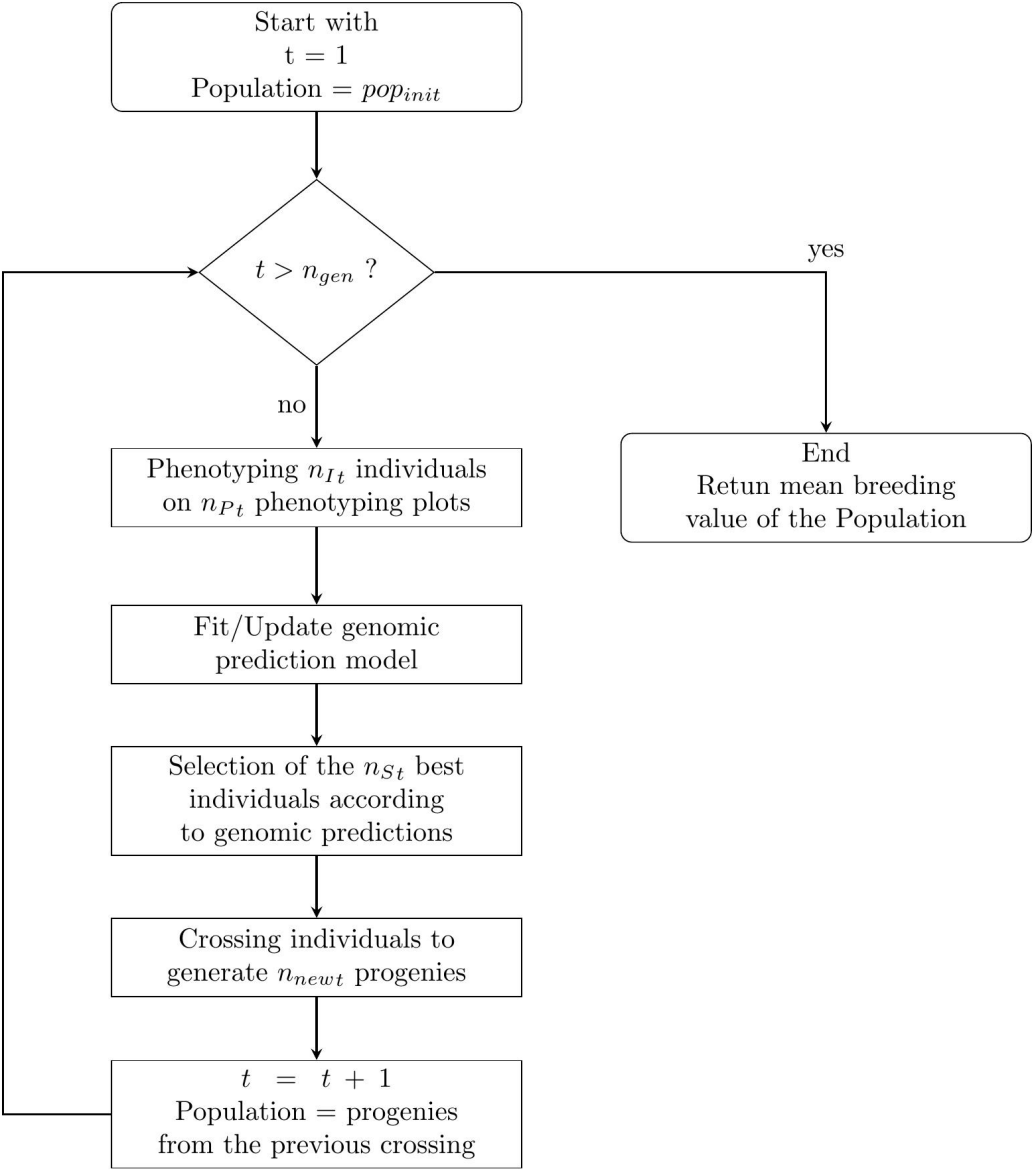
A simple flowchart of the breeding simulation algorithm. The process start with the individuals of the initial population of the breeder ***pop_init_***.

The breeding process was simulated as follows and is summarised in **Figure 1**.

For each breeding cycle *t*∈{1, *n*{_*gen*_}}, we have the following procedure. Note that the *floor* function takes a real number *x*, as input, and returns the greatest integer less than or equal to *x* as the output.

- Phenotyping: To phenotype the *n_I_*t*__* individuals in the *n_P_*t*__* phenotyping plots, we uniformly allocate the as much plots as possible for each individuals. If there are any remaining plots, we randomly select some individuals which will be phenotype one more time. Then we simulate the phenotyping experiments 158 accordingly (see. 2.3.2).
- Genomic prediction: If *n*_*P*_*t*__ ≠ 0 we fit a prediction model using a ridge regression (Friedman et al., 2010) with genome-wide marker genotypes as input variables. The regression model is trained with all available phenotypic and genotypic data collected from the first generation.
- Selection: The *n_S_*t*__* individuals with the highest predicted phenotypic values according to the prediction are selected as parents for the next generation.
- Crossing: The selected parents are mated according to their genetic distances from each other. If *n_S_*t*__* ≥ 4, we use a “Traveling Salesperson Problem” (TSP) algorithm (Croes, 1958). Because TSP algorithms minimize the path linking several cities, we use such algorithm to generate a sequence of individuals *Ind*_*s*_, *s*∈{1, *n*{_*S*_*t*__}} such as,

(4) $$\sum_{s=1}^{n_{S}t-1}\frac{1}{dist\left(Ind_{s},Ind_{s+1}\right)}+\frac{1}{dist\left(Ind_{n_{S}t},Ind_{1}\right)}$$

is small compared to other possible sequences, with dist (*A*, *B*), the Euclidean distance of vectors of the marker genotype scores between individuals *A* and *B*, weighted by the estimated effect of each marker according to the latest prediction model. A mating table is generated associating ∀*s *∈{1, *n*{_*S*_*t*__−1} *Ind*_*s*_ with *Ind_s_*_+1_ and *Ind_nS_*t*__* with *Ind*_1_. If 2 ≤ *n_S_*t*__* ≤ 3, a mating table is generated associating all individuals together, and if *n_S_t__* = 1, the selected individual is self-fertilized. The mating table, composed of *n_C_t__* crosses, is used to generate a total of *n_new_t__* offspring. To calculate the number of progenies for each crosses, we uniformly allocate the total number of offspring to each crosses. If there are any remaining progenies, some crosses are randomly selected to generate one more individual. The new individuals are then generated using a crossing simulation algorithm (cf. 2.3.3).

The next breeding cycle began by considering only the individuals generated from the crosses of parents selected in the previous cycle for phenotyping and selection: ∀*t *∈{2, *n*{_*gen*_, *n*_*I*_*t*__=*n*_*new*_*t*−1__}}.

#### 2.3.2 Phenotyping simulations

In this section, we explain how the phenotypes of individuals were simulated. For simplicity, we ignored the main environmental effects caused by years and locations as well as the effects of genotype-by-environment (G × E) interactions. The model used to simulate the phenotypes was as follows:


(5) $$y_{sr}=G_{s}\beta +e_{sr}$$

where *y_sr_* is the phenotype of individual *s* repetition *r*, *G_s_* is a vector of the genotype of individual *s* encoded with an allele dose (i.e., 0, 1, or 2) for the reference alleles, *β* is the genetic effect of the reference alleles, and $e_{sr}∼N\left(0,\sigma_{e}^{2}\right)$ . The values of these parameters were obtained before the optimisation. The values used in this study are described in Section 2.4.1.

#### 2.3.3 Crossing simulations

To generate the single nucleotide polymorphism (SNP) genotypes of new individuals from those of the parents, we simulated gametogenesis, one from each parent:

For each pair of chromosomes:

1. We draw the number of crossing-overs *n_co_* for each chromosome in a Poisson distribution of rate *λ_chr_*, which is the length of the chromosome in the unit of Morgan: *n_co_* ~ Pois(*λ_chr_*)
2. When *n*_*co *_≠ 0, we draw the positions of crossing-over independently in a uniform distribution along the length of the chromosome in Morgan. We then include the sampled positions *COpos_k_* (∀*k*∈{0, *n*{_*co*_}+1)} of the crossing-overs so that *COpos*_*k *_< *COpos*_*k*+1_ (∀*k *∈{0, *n*{_*co*_}}) with *COpos*_0_ = 0 and *COpos*_*n*_*co*_+1_=*λ*_*chr*_.
3. Let *X* be the 2 × *n_snp_l__* matrix representing the genotype of the parent for the current pair of chromosomes *l*. Each row represents one chromosome of the pair. Let *Y* be the vector of length *n_snp_l__* representing the recombined genotype of the gamete for the chromosome *l*. We set [*a,b*] = [1,2] or [2,1] with probability $\frac{1}{2}$ . *Y* is then calculated as:

$$Y\left[h\right]=\{\begin{array}{ll} X\left[a,h\right] & \text{if }\exists \;k\;\in \left[0,floor\left(\frac{n_{co}}{2}\right)\right],COpos_{2k}\leq pos_{h}<COpos_{2k+1}\\ X\left[b,h\right] & \text{if }\exists \;k\;\in \left[1,floor\left(\frac{n_{co}}{2}\right)\right],COpos_{2k-1}<pos_{h}\leq COpos_{2k} \end{array}$$

where *pos_h_* is the position of marker *h*.

The genotypes of the offspring were obtained by merging two gametes from their parents.

#### 2.3.4 Calculation of the breeding simulation parameters

As mentioned in the section 4.3.1, we used a different parametrisation for the breeding simulation than those used for the optimisation problem. In this section, we explain the derivation of the parameters of the simulation from the constraints and optimised parameters *B*, *C*_*P*_, *C*_*n*_, *n*_*gen*_, *i*_*init*_, *i*,*B*_*rep*_, *pheno*_*p*_.

The breeding process detailed in 4.3.1 uses the following parameters:

- *n_P_t__* the number of phenotyping in the *t*-th generation.
- *n_S_t__* the number of selected individuals in the *t*-th generation.
- *n_new_t__* the number of new individuals to create in the *t*-th generation.

The value of *n_S_t__*, the number of selected individuals in the generation *t*-th, is calculated as the number of individuals in the *t*-th generation multiplied by the selection intensity, *i_init_* for the initial population and *i* for the later, and rounded to the nearest strictly positive integer.

To calculate *n_P_t__* and *n_new_t__*, we first needed to calculate the total number of phenotyping plots available for all generations *n_P_tot__* and the total number of new individuals created during the entire breeding campaign *n_new_tot__* according to the phenotyping and new individual generation costs (*C_p_* and *C_n_*) as well as the total budget *B*.

*n_new_tot__* is calculated by the budget allocated to the generation of new individuals *B* × *B_rep_* divided by the cost of creating a new individual *C_n_*, rounded to the nearest integer.

*n_P_tot__* is calculated by the remaining budget *B* − *n_new_tot__* ×*C_n_* divided by the cost of phenotyping one plot *C_p_*, rounded down to the nearest integer.

Owing to rounding operations, the effective budget used *B*_*eff *_= *n*_*new*_*tot *__× *C*_*n *_+ *n*_*P*_*tot *__× *C*_*P*_ may differ from the given total budget constraint *B*. However, this difference is smaller than *C_P_* which should usually be relatively small with respect to *B*.

We can now calculate *n_P_t__*, the number of phenotyping plots in the *t*-th generation. First the generations which will include phenotyping trials are identified. These are the first generation *t* = 1 and then every *pheno_p_* generation (eg. [1,3,5,…] for *pheno_p_* = 2; [1,4,7,…] for *pheno_p_* = 3). All the other generation will have *n_Pt_* = 0. Consequently, the total number of generations with non-zero phenotyping trials is equal to *n_gen_*/*pheno_p_* rounded up to the nearest integer. For thosegenerations, we equally allocate the total number of phenotyping plots available for the breeding campaign. If this number is not an integer, as many generations as the remaining plot are selected and those generations will be allocated one more phenotyping plot. Moreover, if *n_P1_* < 3, its value is increased to 3 to avoid errors during creation of the prediction model.

Finally, we calculate the number of new individuals to create at each generation. The first generation was homozygous, and therefore, any individuals derived from the same pairs of parents would have the same genotype; as a result, *n_new1_* was set to the number of crosses proceeding at this generation *n_C1_*. Then the remaining number of new individuals to be created in the later generations were allocated equally. If this number is not an integer, as many generations as theremaining new individuals to be created are randomly selected and those generations will generate one more individual.

#### 2.3.5 Implementation, datasets, and simulation parameters

All calculations were performed using R programming language (version 4.0.2) (R Core Team, 2020). A repository containing the code used in this study can be found at GitHub (https://github.com/ut-biomet/bayesianOptimizationForBreeding). The simulation algorithm developed for this study was integrated in the R package *“breedSimulatR”* (Diot and Iwata, 2020), the Bayesian optimisations were performed using the package *“mlrMBO”* (Bischl et al., 2017). An exhaustive list of all packages and their versions used for calculation can be found in the GitHub repository for this study in the file *renv.lock *(https://github.com/ut-biomet/bayesianOptimizationForBreeding/renv.lock).

### 2.4 Parametrisations of scenarios

To test our optimisation method, we ran several optimisations following the algorithm detailed in the “Materials and methods” section with different parameterisations.

#### 2.4.1 Simulation setup

The genotypes of the initial population were created based on the whole-genome sequences of the accessions of soybean mini-core collections provided by the National Agricultural Research Organisation, Japan (Kaga et al., 2012) (Kajiya-Kanegae et al., 2021)[4.4.1]. These data represent 198 accessions of soybean (*Glycine Max*) with a total of 4,776,813 SNP markers on 20 pairs of chromosomes. To make the simulations faster, the genotypes of the initial population consisted of a smaller subset of *n_snp_* = 3000SNPs that were randomly selected. For simplicity, we arbitrarily set the chromosome length to 1 Morgan (i.e. 100 cM) and calculated the linkage map positions based on physical positions, assuming a linear relationship between the two types of positions. This led to an average of one crossing over for each chromosome during the gametogenesis simulation.

The true genetic effects of the SNPs *β* on the phenotypic traits were determined as follows. First, we selected a subset of *n_qtn_* =1000 SNPs in the pool of all available *n_snp_* SNPs in the genotypes of the initial population. These markers have non-null effects and are known as quantitative trait nucleotides (QTNs). Let *β_m_* be the effect of the QTN *m*, for all *m*∈{1, *n*{_*qtn*_}}, *β_m_* was drawn according to the formula *β_m_* = *a_m_* × *I* with *a_m_* following an exponential distribution, *a_m_* ~ *Exp*(*λ* = 1) and *I* was equal to +1 or −1 with probability $\frac{1}{2}$ . The effects of the *n_sn_ − n_qtn_* remaining markers were set to 0.

The residual variance of the simulated phenotypes *σ_e_* was calculated according to the specific heritability for the initial population $H_{0}^{2}$ . As only the additive genetic effects were simulated in this study, $H_{0}^{2}$ represented both the narrow and broad sense heritability in the initial population. Heritability is the ratio between genotypic variance and phenotypic variance:


$$H_{t}^{2}=\frac{Var\left(G_{t}\beta \right)}{Var\left(y_{t}\right)}=\frac{Var\left(G_{t}\beta \right)}{Var\left(G_{t}\beta \right)+\sigma_{e}^{2}}$$

where *G_t_* is the genotype of individuals from generation *t*, *y_t_* is the phenotype of individuals from generation *t*, and *beta* is the marker effect. We can thus deduce that:


$$\sigma_{e}^{2}=Var\left(G_{t}\beta \right)\left(\frac{1}{H_{t}^{2}}-1\right)$$

The value of $\sigma_{e}^{2}$ is then calculated using the values of *Var*(*G_t_β*) and $H_{t}^{2}$ for the initial population *t = 0*. Thus, $\sigma_{e}^{2}=\overset{n_{I1}}{\underset{s=1}{\sum }}\frac{{\left(G_{0}\beta -\bar{G}\beta \right)}^{2}}{n_{I1}-1}$ , where $\overset{-}{G_{0}\beta }$ denotes the average genetic value of the initial population. In this study, we set $H_{0}^{2}=0.7$ and $H_{0}^{2}=0.3$ .

#### 2.4.2 Constraint parameters

We considered the constraints related to the total number of selection cycles in the breeding program and the total budget for the program. To test the proposed optimisation method on various breeding schemes, we used setups with a total number of selection cycles for breeding campaigns of *n_gen_* = 5 and *n_gen_* = 10. For simplicity, both the costs for phenotyping one plot *C_p_* and for generating and phenotyping, one new individual *C_n_* were set as one and two total budgets were tested: *B* = *n_gen_* × 200*C_p_* and *B* = *n_gen_* × 600*C_p_*.

All possible combinations of these parameterisations were tested, and yielded eight different scenarios:


(a) $$H^{2}=0.3,n_{gen}=5,B=5\times 200$$

(b) $$H^{2}=0.3,n_{gen}=5,B=5\times 600$$

(c) $$H^{2}=0.3,n_{gen}=10,B=10\times 200$$

(d) $$H^{2}=0.3,n_{gen}=10,B=10\times 600$$

(e) $$H^{2}=0.7,n_{gen}=5,B=5\times 200$$

(f) $$H^{2}=0.7,n_{gen}=5,B=5\times 600$$

(g) $$H^{2}=0.7,n_{gen}=10,B=10\times 200$$

(h) $$H^{2}=0.7,n_{gen}=10,B=10\times 600$$

#### 2.4.3 Parametrisation of optimisation

The initial training data for Bayesian optimisation are a set of five points randomly sampled in a Latin hypercube (Beachkofski and Grandhi, 2002); we set the minimal distance between two sampled points as *filter_tol_* = 10^-3^

The random optimisation algorithm does not require training data; however, to obtain the same number of objective function evaluations as those with Bayesian optimisation, we began its first iteration with five points randomly selected in the parameter space.

We ran two batches of optimisation. For the first batch, with each scenario and each optimisation method, we repeated 16 optimisation runs with *n_iter_* = 50 iterations of *q* = 8 parallelly sampled points at each iteration. After each optimisation run, *n_evaluation_* = 32 breeding schemes were repeated using the parameters returned by the optimisation run. The results from these optimisations were used to compare the behaviour of Bayesian optimisation against random optimisation.

However, we conducted these optimisations independently. Thus, there was no direct association between each run of Bayesian optimisation and random optimisation (for each scenario). To compare the runs of Bayesian optimisation and random optimisation, such an association is required. To be as representative as possible in our comparisons, for each method and run, we calculated the cumulative maxima of the objective function values over the optimisation iterations. Subsequently, we calculated the average cumulative maxima for each method. Finally, to represent one method, we chose the run that was closest (in terms of mean square error) to the average cumulative maxima of the method.

For the second batch, in a more pragmatic manner, we tried to mimic what a breeder who would like to use Bayesian optimisation can do with relatively limited computer power and time available for optimisation. We set the number of iterations to *n_iter_* = 15 with *q* = 2 parallel sampling points at each iteration. After each run, we simulated a *n_evaluation_* = 1 breeding scheme using the parameters returned by the optimisation run.

We planned to run these optimisations 1024 times for each scenario and method. Over the 1024 × 8 = 8129 Bayesian optimisations planned, the library used for Bayesian optimisation encountered an unexpected error in 16 cases. Because the number of failed runs was low (approximately 0.2%) and identifying and fixing this bug would require a huge effort, we decided not to consider those runs. This explains why the total number for each optimisation is not exactly 1024 for some scenarios (1017 in the worst case for scenario g.). Moreover, in practical cases, the probability that a user encounters such a bug is quite low; however, in such cases, the user can re-run the optimisation with a different random seed to solve the problem.

The optimisations were conducted simultaneously on two computers, each with 256GB of RAM and an AMD Ryzen Threadripper 3990X @2.9GHz 64 cores CPU. One computer performed the optimisations of all scenarios with *H^2^* = 0.3 and the other with *H^2^* = 0.7. Because one optimisation required *q* cores, 64/*q* optimisations were run in parallel.

## 3 Results

### 3.1 Optimisation behaviour

#### 3.1.1 Example of optimisation progress

Here, we detail the behaviour of the optimisation using examples.

The results presented in this section are obtained from the batch in which the optimisations were repeated 16 times with *n_iter_* = 50 iterations of *q* = 8 parallel-sampled points at each iteration for all scenarios. The figures we present associate the representative runs of Bayesian optimisation with random optimisation, as defined in the Materials and Methods section (see. 2.4.3).

**Figure 2:**
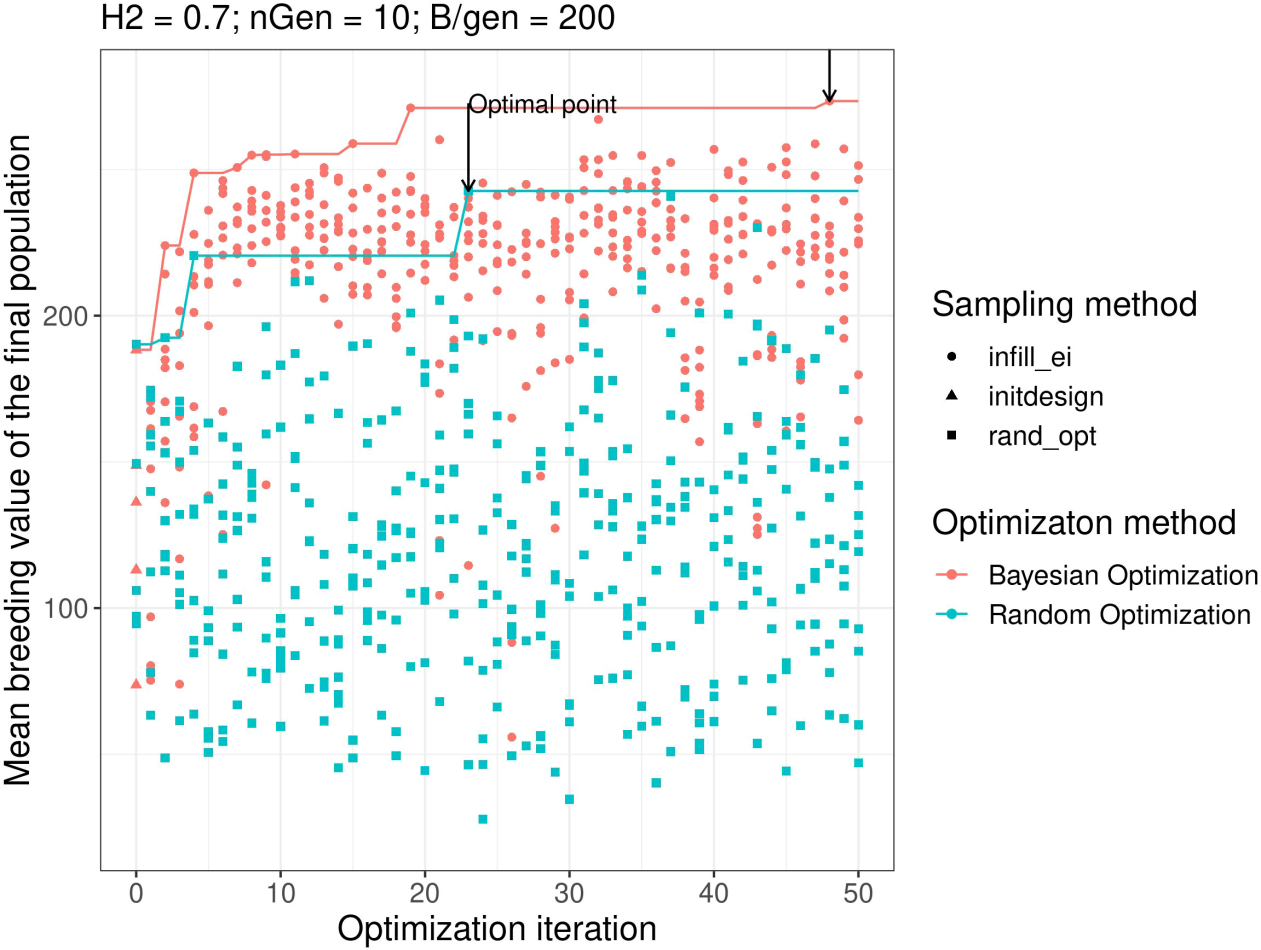
Examples of optimisation progress for the first optimisation parametrisation, scenario ***H^2^*** = **0.3**, ***n_gen_*** = **10**,**B** = **10** × **600**. Lines represent the cumulative maximum over optimisation iterations. *prop.type* is the method used to select the points: *infill_ei*: points proposed by maximizing the EI, *initdesign*: initial sampling points for the Bayesian optimisation, *rand_opt*: points proposed by random optimisation.

**Figure 2** shows the results of objective function evaluations for each optimisation iteration in both Bayesian optimisation and random optimisation for scenario g. As expected, random optimisation explored the points for which the evaluation values of the objective function were evenly distributed across all optimisation iterations. In contrast, Bayesian optimisation yielded results similar to random optimisation for the first iteration but quickly explored points with higher objective function evaluation values. Moreover, Bayesian optimisation continues to explore points with high objective function values.

#### 3.1.2 Explored region of the research space

It is difficult to visualise the parts of the research space that have been explored by optimisation algorithms, because it has four dimensions. To ease this visualisation, we performed principal components analysis (PCA) on the data generated using the optimisations presented in **Figure 2**. In the PCA, we treated the optimised parameters *i*, *i_init_*, *B_rep_*, *pheno_p_* as variables, whereas the points sampled by the Bayesian optimisation algorithm were active individuals (that is,individuals used for calculating the PC axes), and the points sampled by random optimisation were supplementary individuals (that is, individuals, whose scores were calculated based on the axes).

**Figure 3:**
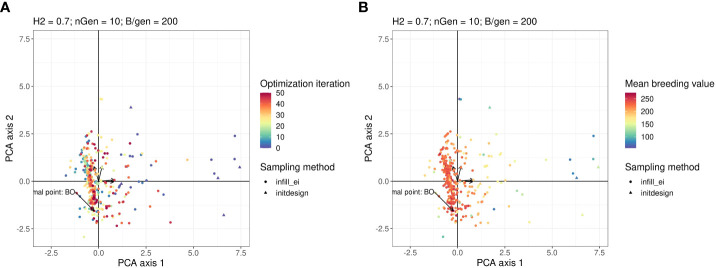
Points in the parameter space explored by the Bayesian optimisation projected on the PCA plan. This plan was calculated using the points explored by the Bayesian optimisation as active individuals. The arrows starting from the origin of the graph are the projections of the active variables. For readability, the variable names associated with each arrow are not written, but this can be found in the Figure in the **Supplementary Material**. Points in the sub-figure **(A)** are coloured according to their iteration and those in the sub-figure **(B)** are coloured according to their corresponding value returned by the objective function.

**Figure 3A** shows a graph projecting the four-dimensional sampling points onto the plane spanned by the first two PCs. The points were coloured according to the number of iterations at which they were explored. Bayesian optimisation explored everywhere in the research space initially and then gradually focused its search on a specific area. As shown in **Figure 3B**, which is the same plot as in **Figure 3A** but with points coloured according to their observed value of the objective function, the objective function tends to have high values in this region with regard to all explored points.

**Figure 4:**
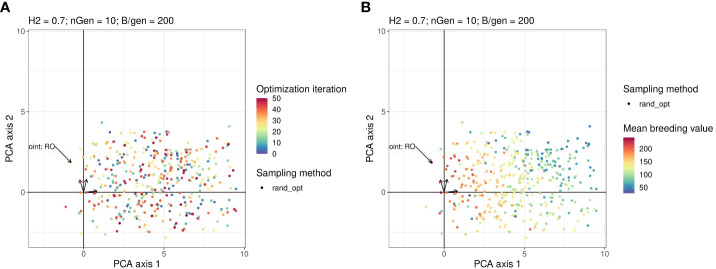
Points in the parameter space explored by the Random optimisation projected on the same plan as in **Figure 3**. The arrows starting from the origin of the graph are the projections of active variables. For readability, the variable names associated with each arrow are not written but can be found on the Figure in the **Supplementary Material**. Points in the sub-figure **(A)** are coloured according to their iteration and those in the sub-figure **(B)** are coloured according to their corresponding value returned by the objective function.

**Figures 4A, B** show the projection of the points sampled by random optimisation of the 2 first principal components in the PCA. This shows that the area explored by the Bayesian optimisation algorithm has not been extensively explored by the random optimisation algorithm.

### 3.2 Comparison between Bayesian optimisation and random optimisation

In this section, we compare for each scenario, the optimised breeding schemes returned by one run of Bayesian optimisation and one run of random optimisation (the ones defined as representative in the Materials and Methods section (see. 4.4.3). Thus, we present the “evaluation results” (see. 4.2.1) returned by the optimisations with *n_iter_* = 50 iterations of *q* = 8 parallel sampled points. (i.e., we focus on the breeding schemes repeated *n_evaluation_* = 32 times using the parameters returned by the optimisation runs).

We present in **Table 1** the number of times Bayesian optimised schemes gave better results (i.e., returned a higher value) than the random optimised schemes across all comparisons (i.e., we compared each of the 32 breeding scheme simulated using the results of the Bayesian optimisation run against each of the 32 breeding schemes simulated the results of random optimisation run; therefore we performed 32 × 32 = 1024 comparisons).

**Table 1:** Proportion of times one specific Bayesian optimised schemes outperformed a random optimised schemes for all scenarios.

Scenario	BO performance
a. *H^2^* = 0.3, *n_gen_* =5, *B* = 5 × 200	83.11%
b. *H^2^* = 0.3, *n_gen_* =5, *B* = 5 × 600	57.32%
c. *H^2^* = 0.3, *n_gen_* =10, *B* = 10 × 200	56.35%
d. *H^2^* = 0.3, *n_gen_* =10, *B* = 10 × 600	75.20%
e. *H^2^* = 0.7, *n_gen_* =5, *B* = 5 × 200	52.25%
f. *H^2^* = 0.7, *n_gen_* =5, *B* = 5 × 600	86.43%
g. *H^2^* = 0.7, *n_gen_* =10, *B* = 10 × 200	73.93%
h. *H^2^* = 0.7, *n_gen_* =10, *B* = 10 × 600	61.33%

Each scheme has been simulated independently using the same parametrization 32 times. “BO performance” column represents the proportion of times Bayesian optimised schemes outperformed the random optimised schemes over all combinations of the 32 simulations (ie. 1024 comparisons) expressed in percentage.

Bayesian optimisation yielded the lowest performance for scenario e. where it was better 52.2% of the time (532 comparisons out of 32^2^), whereas the highest performance was for scenario f. where it was better 86.43% of the time (885 comparisons out of 32^2^).

For half of the scenarios, Bayesian optimisation was better more than 67% of the time.

**Figure 5:**
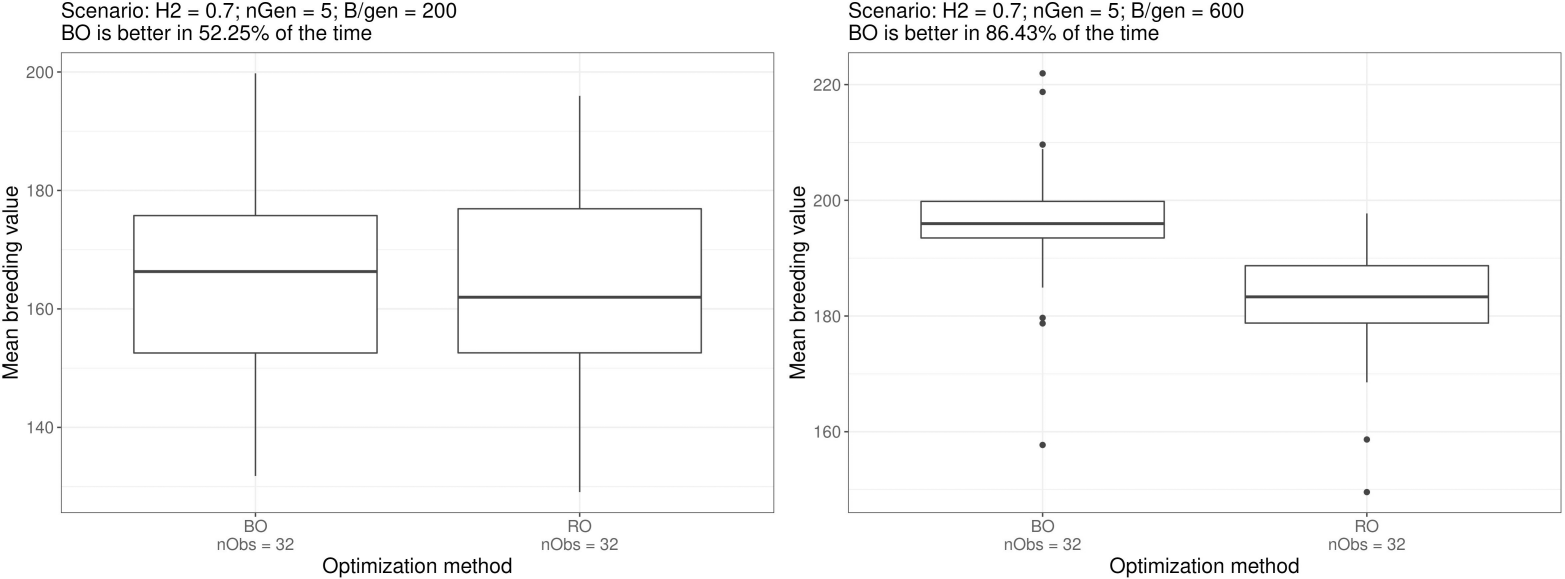
Boxplots of simulation outputs for 32 repeated simulations using the parametrisation suggested by the optimisation method for scenarios e. and f. The number of times Bayesian optimised schemes outperformed the random optimised schemes (among the exhaustive 2 by 2 comparisons) is displayed above the plots. Similar boxplots for all the scenarios can be found in the **Supplementary Materials**.

**Figure 5** shows the boxplots of the breeding simulation results for the Bayesian optimised parameters and random optimised parameters for scenarios e. and f., the two scenarios mentioned above. And the boxplot for all the scenarios can be found in the **Supplementary Materials**.

Next, we compared for each scenario, all optimised breeding schemes returned by all the 1017 to 1024 runs of Bayesian and random optimisations of *n_iter_* = 15 iterations with *q* = 2 parallel sampled points at each iteration. Each optimisation run was evaluated *n_evaluation_* = 1 times (i.e., after each run, we simulated one breeding scheme using the optimised parameters).

As we compared the optimised breeding schemes from different runs of optimisation, the variance of these data was derived from both the stochastic nature of the optimisation algorithms and the stochastic nature of the simulated breeding campaigns. This reflects what a breeder would face by performing one optimisation and then one breeding scheme.

**Table 2** shows the proportion of outcomes where the Bayesian optimisation results yielded better results (i.e., returned a higher value) than random optimisation over all the ∼ 1017^2^ = 1034289 to ∼ 1024^2^ = 1048576 comparisons (all the 1017 to 1024 breeding schemes simulated after Bayesian optimisation runs compared against the 1017 to 1024 simulated breeding schemes after the random optimisation runs), Bayesian optimisation showed the lowest performance for scenario a. where it was better in 57% of the cases and was the best for scenario f. where it was better in 75% of the cases. For half of the scenarios, the Bayesian optimisation results were better in more than 65.6% of the cases.

**Table 2:** Proportion of times Bayesian optimised schemes outperformed random optimised schemes for all scenarios.

Scenario	Number of optimisations	BO performance
a. *H* ^2^=0.3,*n*_*gen*_=5,*B*=5×200	1023	56.99%
b. *H* ^2^=0.3,*n*_*gen*_=5,*B*=5×600	1024	66.24%
c. *H* ^2^=0.3,*n*_*gen*_=10,*B*=10×200	1020	58.33%
d. *H* ^2^=0.3,*n*_*gen*_=10,*B*=10×600	1024	65.23%
e. *H* ^2^=0.7,*n*_*gen*_=5,*B*=5×200	1020	62.63%
f. *H* ^2^=0.7,*n*_*gen*_=5,*B*=5×600	1024	74.90%
g. *H* ^2^=0.7,*n*_*gen*_=10,*B*=10×200	1017	65.88%
h. *H* ^2^=0.7,*n*_*gen*_=10,*B*=10×600	1024	73.24%

Simulations have been run 1 time for each of the 1017 to 1024 optimisations for both Bayesian optimisation and random optimisation.”Numberof optimisations” column represents the number of optimisations actually performed for each optimisation method. “BO performance” column represents the proportion of times Bayesian optimised schemes outperformed the random optimised schemes over all combinations (ie. between **1017^2^** and **1024^2^** comparisons) expressed in percentage.

**Figure 6:**
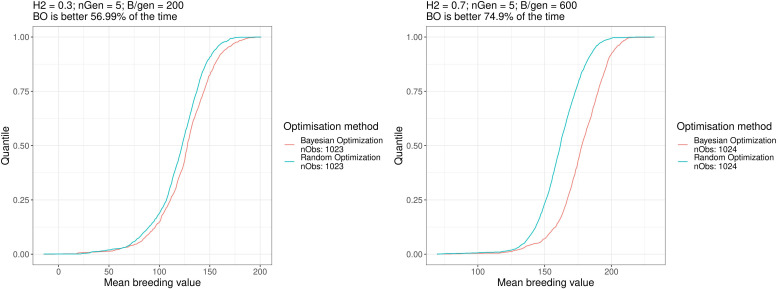
Empirical distribution functions of the breeding simulation results parametrised using the results of Bayesian optimisation and random optimisation for scenarios a. and f. Similar plots for all the scenarios can be found in the **Supplementary Materials**.

**Figure 6** shows the empirical cumulative distribution functions (ECDFs) of the results for both optimisation algorithm for scenarios a. and f., the two scenarios mentioned above. In these plots, the horizontal axis shows the results of the simulated breeding schemes, and the vertical axis shows the quantiles over all repetitions. The point on the horizontal axis, which has quantile 0.5, is the median value of the data. As such, the further toward the right and bottom directions acurve is, the better the method is. These ECDFs are available for all the scenarios in the **Supplementary Materials**. Overall, the Bayesian optimisation method performed better than random optimisation for all scenarios.

### 3.3 Distribution of the optimised parameters

**Figure 7:**
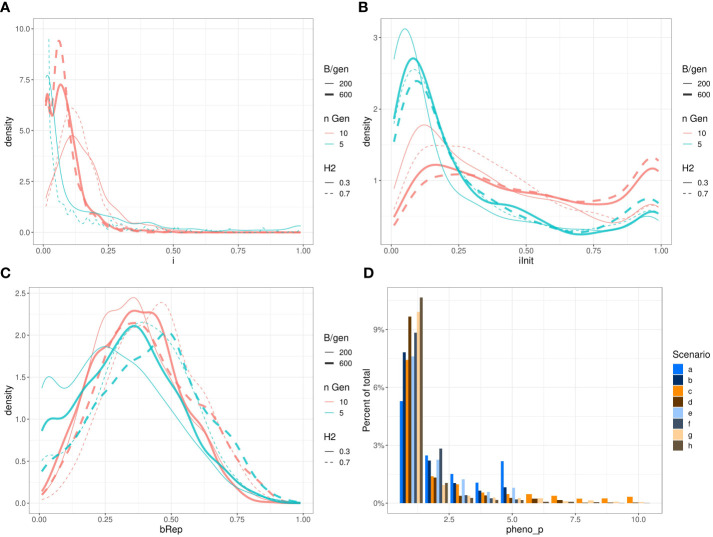
Marginal distributions of the optimised parameters *i* **(A)**; *i_Init_* **(B)**; *B_rep_* **(C)** and *pheno_p_* **(D)** returned by Bayesian optimisation. To keep the subfigure **(A)** readable, we have removed the scenarios b. and f. (*n_gen_* = 5 and *B* = *n_gen_* × 600). Those densities stick on the left side of the graph and raised to very high values: respectively ~150 and ~800. Also, we restricted the vertical axis from 0 to 10 therefore, the top of the curve for scenario e. (*H* ^2^=0.7,*n*_*gen*_=5,*B*=5×200 ) reaching ~50 is out of bound.

**Figure 7** presents the observed marginal distributions of the optimised parameters returned by all the ~1024 runs of Bayesian optimisations of *n_iter_* = 15 iterations with *q* = 2 parallel sampled points at each iteration for each scenario. These marginal distributions do not have simple shapes and differ from scenario to scenario. For example, the distributions of the parameter *i_Init_* are bi-modal and relatively flat for some scenarios, and the distributions of the parameter *i* have different expected values.

**Figure 8:**
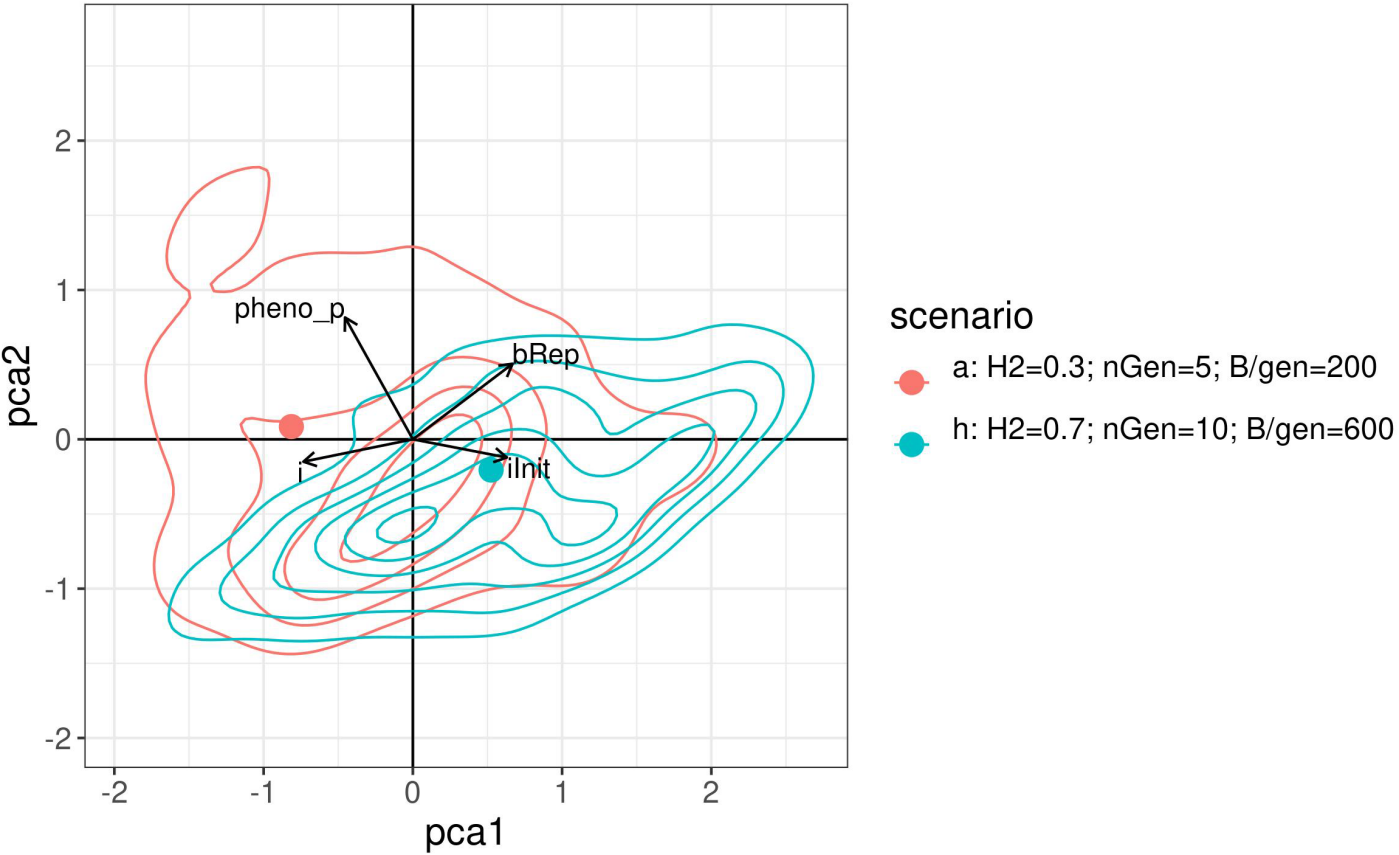
2 dimensional density plot of principal component analysis projection of the optimised parameters returned by the Bayesian optimisation for scenarios a. and h. The points represent the projection of the centre of gravity for the points cloud of each method.

Visually compare the joint distributions of the optimised parameters is not possible as it would require four dimensions. Therefore, a PCA was performed on the optimised parameters of the above results. **Figure 8** shows a graph projecting the four optimised parameters onto the plane spanned by the first two PCs. For readability, this figure shows only the results for scenarios a. and h. with contour lines for the densities. The two points represent the projection of the centre of gravity for the point.

Like the empirical marginal distributions, the distribution shapes were found to differ for the two scenarios, and to be quite complex. This may suggest a complex covariance structure between the optimised parameter which may also depend on the constraints.

## 4 Discussion

In this study, an optimisation method applied to a simulated breeding scheme was described and tested. Even if, by its design, Bayesian optimisation cannot ensure finding the global maximum, it can rapidly find breeding scheme parameters that yield good results regarding the entire parameter space. Further, Bayesian optimisation outperformed a naive optimisation method.

Moreover, the empirical distribution of the optimal parameters found using Bayesian optimisation differed according to the constraints applied, even if the constraints and breeding parametrisation used were relatively simple. Additionally, the shape of those distributions may suggest that some breeding parameters may be more or less important depending on the breeding constraints, and that several local optima may be present which makes this optimisation difficult. This supports the work of (Henryon et al., 2014) and highlights the importance of considering the constraints during the design of an optimal breeding scheme. This last point is particularly important because breeders have different available resources, so a “case by case” approach to optimise breeding schemes is advisable.

The method is also quite flexible, and if a breeding simulation algorithm can provide an objective function, Bayesian optimisation algorithms can theoretically be applied. Currently, there are several computing libraries and software that can ease the creation of breeding simulations, including *AlphaSimR* (Gaynor et al., 2020), *BreedingSchemeLanguage* (Yabe et al., 2017), and *BreedSimulatR* (Diot and Iwata, 2020). Therefore, breeders can apply Bayesian optimisation to cater to their specific cases. This also allows the user to easily test several simulation algorithms using different genetic architectures.

The optimisation algorithm itself can be adapted for practical use. In this test, because of the nature of the analysis, we used a fixed number of iterations as the stopping criterion for optimisation. However, other criteria, such as ending the optimisation after a specific time or when a set of parameters returns a value above a specific target can also be used.

The proposed approach still has some limitations. First, the breeding scheme parametrisation and constraints presented in this paper are simple, and we used only four numeric parameters for parametrisation of the breeding scheme. Moreover, only one selection criterion and one mating method were used, which were not part of the scheme parameters. Further, most generations had the same selection intensity, and there were no constraints on the number of phenotyping or genotyping at each generation. Further investigations should thus be conducted with more complex breeding scheme parametrisation to obtain insights into the robustness of this method. Such studies will require the use of categorical parameters, which could be more difficult to include in a Bayesian optimisation framework, because they usually assume a continuous change in the value of an objective function (Zhang et al., 2020b) (Zhang et al., 2020a). Moreover, Bayesian optimisation is known to not perform well in a research space with more than 20 dimensions (Frazier, 2018) (Moriconi et al., 2020). Because the Gaussian process relies on the distances between points to calculate the kernel, it is sensitive to the “curse of dimensionality” (Chen, 2009). Additionally in high dimensions, the acquisition function becomes flat with few peaks which make the usage of global optimization algorithm unfeasible (Rana et al., 2017).

Second, optimisation uses breeding simulations rather than actual breeding campaigns. Therefore, it is not possible to guarantee that the results are valid for actual breeding. These results depends on how well the implemented simulation reflects reality.The main sources of divergence can be derived from the simulation algorithm itself and the information used.

For example, during our breeding simulation, even if the ßimulated breeder” does not know the true marker effects, all the QTNs related to the phenotypic trait of interest are available in the genetic data used to build the genomic prediction models, which is usually not the case in actual breeding campaign. Additionally, the genetic architecture and probability distributions implemented in the simulation are not the same as those in reality. All the parameters of the simulation like the “real marker effects” and “real linkage map position” may not be known by the user. One could still use values based on estimates and assumptions derived from empirical information, but those estimations can be noisy and/or biased and their effects on the accuracy of thesimulation are not yet known.

## 5 Conclusions

This study is one of the first to apply Bayesian optimisation to the design of breeding schemes while considering constraints. The presented approach has some limitations and should be considered as a first proof of concept that demonstrates the potentialof Bayesian optimisation when applied to breeding schemes. It also presents the integration of breeding constraints into the breeding scheme design in aims of optimisation which can provide a basis for further research. Bayesian optimisation applied to simulated breeding campaigns may provide a new tool to breeders that, rather than providing strict guidelines, may give insight to design new breeding schemes. For example, a practical application could be to estimate some marker effects for a particular phenotypic trait, phase the genotypes of a population, write the simulation function of a potential breeding scheme under some constraints, and finally optimize this function. The results returned by the optimizer could be an interesting line of thought when compared with the intuition of the breeder or their expectations. This may provide information which can assist the breeder in making decisions on designing breeding programs.

Since the code we wrote for this article is freely available online (Diot and Iwata, 2022), and we did our best to make the BreedSimulatR (Diot and Iwata, 2020) R-package intuitive and easy to use, anyone with some knowledge with R programming language (R Core Team, 2020) can reproduce, extend or adapt our 471 method on their particular cases.

Further studies, focusing on the sensibility of the presented approach regarding the errors brought by the assumptions of the simulations, would be necessary to improve the confidence on its usage in a more practical manner.

## Data availability statement

The data analyzed in this study is subject to the following licenses/restrictions: This data-set can potentially be shared on request to the authors. Requests to access these datasets should be directed to Hiroyoshi IWATA, hiroiwata@g.ecc.u-tokyo.ac.jp.

## Author contributions

JD developed the simulation algorithm, wrote the optimisation code, created the figures, and drafted the manuscript. HI conceived and designed the study, provided administrative support, and supervised the study. Both authors read and approved the final manuscript.
