# Supplementary material for: Bayesian optimisation for breeding schemes
Source: Front Plant Sci. 2023 Jan 11;13:1050198. doi: 10.3389/fpls.2022.1050198 (PMC9875003; doi:10.3389/fpls.2022.1050198)
Supplement: Supplementary file 1 [file DataSheet_1.pdf]

Supplementary Material

1 SUPPLEMENTARY TABLES AND FIGURES

1.1 PCA related graphs

See figure S.1.

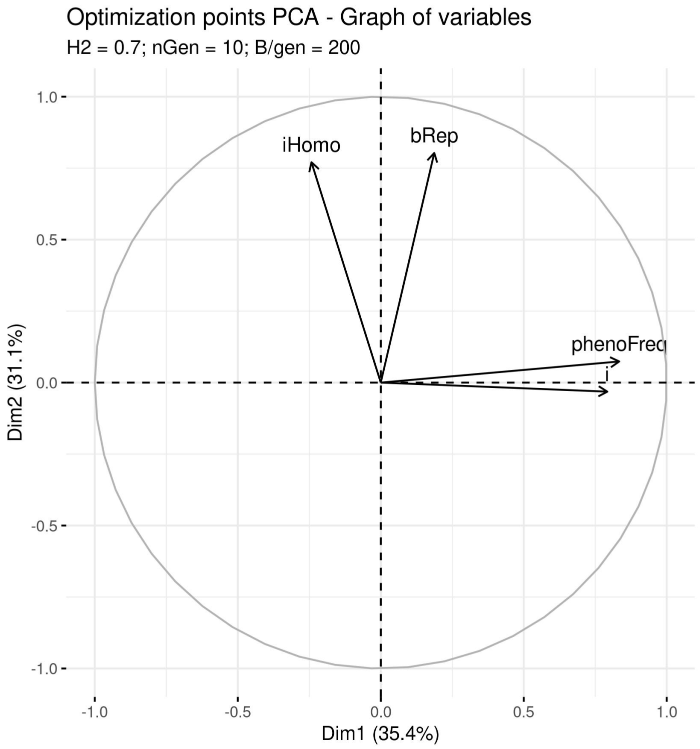

Figure S.1. Graph of the active variables used for the principal component analysis (PCA) in figures **3** and **4**

1.2 Comparison between BO optimized schemes and RO optimized schemes

See figure S.2.

1.3 Empirical cumulative distribution functions

See figure S.3.

1.4 Source code and data

The source code and generated data related to the presented analysis are available online at `https://github.com/ut-biomet/bayesianOptimizationForBreeding`.

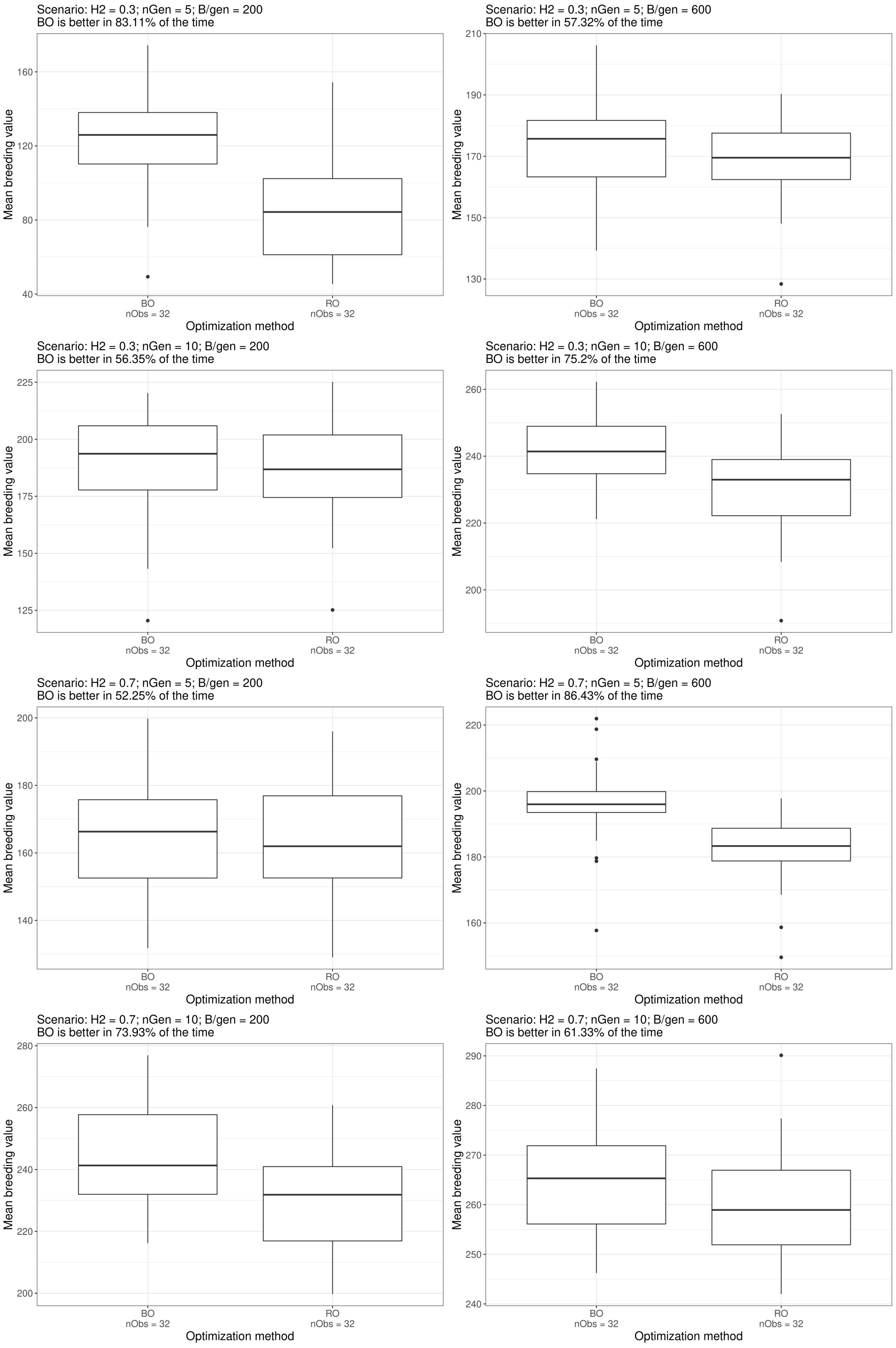

Figure S.2. Boxplots of simulation outputs for 32 repeated simulations using the parametrisation suggested by the optimisation method for all scenarios. The number of times Bayesian optimised schemes outperformed the random optimised schemes (among the exhaustive 2 by 2 comparisons) is displayed above the plots.

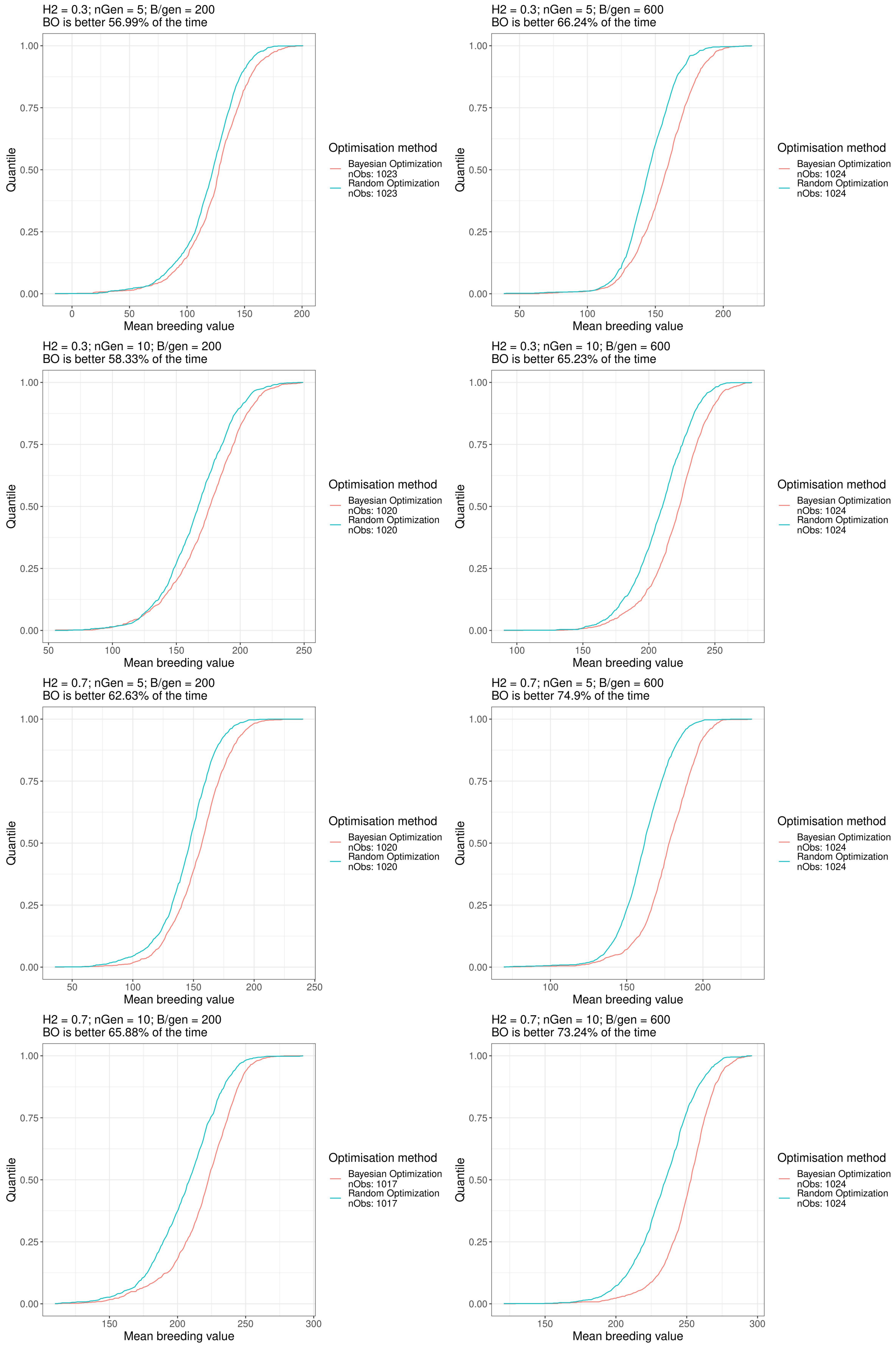

Figure S.3. Empirical distribution functions of the breeding simulation results parametrised using the results of Bayesian optimisation and random optimisation for each scenario.
